# CAPE increases the expression of SOD3 through epigenetics in human retinal endothelial cells

**DOI:** 10.3164/jcbn.16-109

**Published:** 2017-06-20

**Authors:** Atsuko Ohashi, Hiroyuki Yasuda, Tetsuro Kamiya, Hirokazu Hara, Tetsuo Adachi

**Affiliations:** 1 Department of Biomedical Pharmaceutics, Laboratory of Clinical Pharmaceutics, Gifu Pharmaceutical University, 1-25-4 Daigaku-nishi, Gifu 501-1196, Japan

**Keywords:** extracellular-superoxide dismutase, caffeic acid phenethyl ester, diabetic retinopathy, histone deacetylase, myocyte enhancer factor 2

## Abstract

Extracellular-superoxide dismutase (EC-SOD or SOD3), which catalyzes the dismutation of superoxide anions into hydrogen peroxide, plays a key role in vascular protection against reactive oxygen species (ROS). The excess generation of ROS is closely involved in the pathogenesis of diabetic retinopathy (DR); therefore, the maintenance of SOD3 expression at high levels is important for the prevention of DR. In the present study, we showed that caffeic acid phenethyl ester (CAPE) increased the expression of SOD3 through the acetylation of histone within the *SOD3* promoter region in human retinal endothelial cells (HRECs). Histone acetylation within its promoter was focused on the inhibition of histone deacetylase (HDAC), and we examined the involvement of myocyte enhancer factor 2 (MEF2) and HDAC1 in CAPE-elicited SOD3 expression. Our results demonstrate that SOD3 silencing in basal HRECs is regulated by HDAC1 composed with MEF2A/2D hetero dimers. Moreover, phosphorylation of threonine 312 in MEF2A and dissociation of HDAC1 from *SOD3* promoter play pivotal roles in CAPE-elicited SOD3 expression. Overall, our findings provide that CAPE may be one of the seed compounds that maintain redox homeostasis.

## Introduction

Diabetes, a metabolic condition characterized by high blood glucose levels, is a well-known lifestyle-related disease. Sustained hyperglycemia leads to the progressive development of long-term complications that affect the macrovascular and microvascular systems.^(1,2)^ Diabetic retinopathy (DR) is a potentially blinding complication of diabetes, and is characterized by damage to the microvasculature of the retina.^(3)^ Oxidative stress is known to be enhanced under hyperglycemic conditions and is one of the causes of the progression of DR;^(4)^ therefore, the proper maintenance of the antioxidant system is important for the amelioration of oxidative stress, and as a consequence, the progression of DR.

Superoxide dismutase (SOD), a major antioxidative enzyme, protects cells from the damaging effects of superoxide by accelerating the dismutation reaction of superoxide. Three types of SOD isozymes are present in mammals; copper and zinc-containing SOD (Cu,Zn-SOD or SOD1), manganese-containing SOD (Mn-SOD or SOD2), and extracellular-SOD (EC-SOD or SOD3). SOD3 is a secretary glycoprotein that exists in extracellular spaces and contributes to maintaining redox homeostasis in the vascular system. Previous studies reported that the lack of SOD3 was associated with the pathogenesis of DR.^(5–7)^ Accordingly, the maintenance of SOD3 at high levels may alleviate oxidative stress in the retina and, thus, prevent DR.

**Fig. 1:**
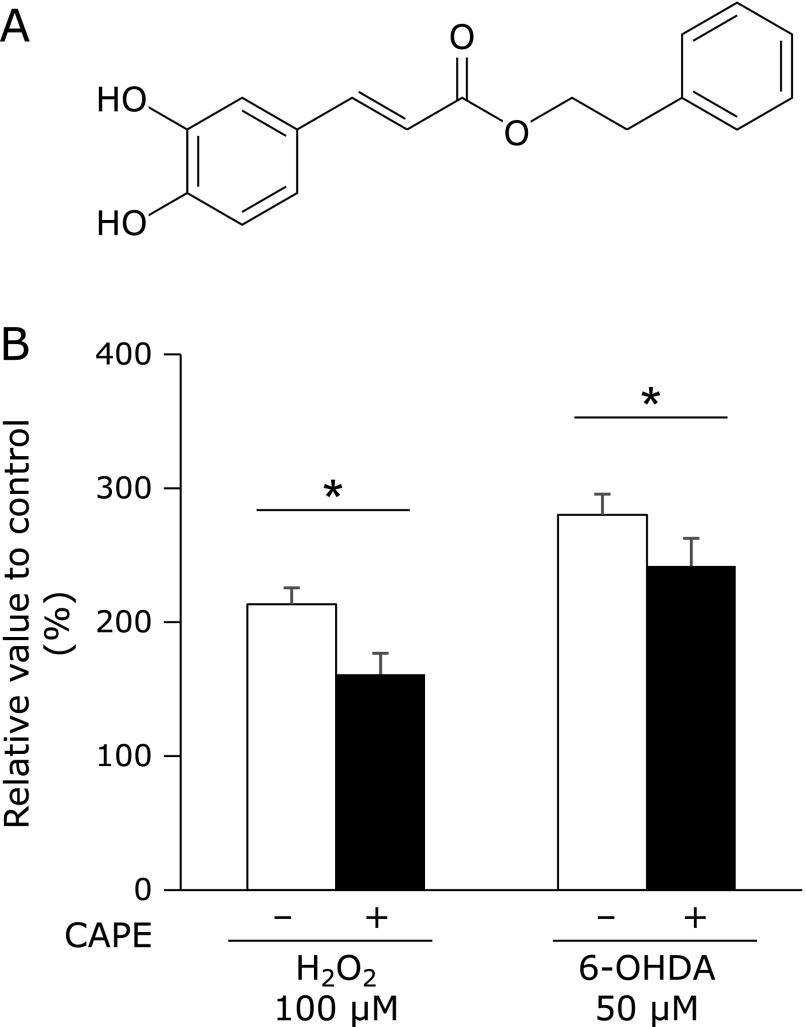
Effects of CAPE on oxidative stress-triggered HRECs injury. (A) Structural formula of CAPE. (B) HRECs were pretreated with 10 µM CAPE for 1 h, and then treated with 100 µM H_2_O_2_ or 50 µM 6-OHDA for 24 h. After cells had been treated, LDH activity in conditioned media was measured. Data were shown as the mean ± SD (*n* = 4). *p<0.05.

Caffeic acid phenethyl ester (CAPE), shown in Fig. 1A, is acquired from propolis and exhibits several bioactivities such as anti-cancer, anti-oxidant, and anti-inflammatory activities.^(8,9)^ CAPE has been shown to exert epigenetic effects and regulates oncogenic gene activity as well as the expression of tumor-suppressor genes.^(10)^ Epigenetics is defined as heritable changes in gene activity and expression that occur without alterations in DNA sequences.^(11)^ These non-genetic alterations are tightly regulated by two major epigenetic modifications: DNA methylation and histone modifications.^(12)^ DNA methylation occurs at the 5' position of cytosine within CpG, and is associated with transcriptional gene silencing.^(13)^ On the other hand, the acetylation of histone at the ε-*N*-terminal has been shown to induce transcriptional activation, whereas the methylation and phosphorylation of histones cause transcriptional activation or repression depending on the position of the modified residues.^(12,14,15)^ Histone acetylation is regulated by the fine balance between histone acetylating and deacetylating enzymes; histone acetyltransferases (HATs) add, whereas histone deacetylases (HDACs) remove an acetyl group. The degree of histone acetylation plays a crucial role in chromatin remodeling and also in the regulation of gene transcription.^(16)^ We previously demonstrated that the up-regulation of SOD3 by exendin-4, a glucagon-like peptide receptor agonist, in human retinal endothelial cells (HRECs) was stimulated by histone acetylation.^(17)^

Myocyte enhancer factor 2 (MEF2) belongs to the MCM1, agamous, deficients, and SRF (MADS) family of transcription factors, and four MEF2 genes (MEF2A, B, C and D) are expressed in distinct, but overlapping patterns in embryonic and adult tissues. MEF2 has been shown to influence the expression of numerous genes depending on and in cooperation with other transcription factors including protein phosphatase 1α (PP1α), β-catenin, and myoblast determination protein (MyoD).^(18–21)^ Moreover, the activity of MEF2 may be modulated through protein–protein interactions, notably with HDACs and HATs.^(22–27)^ Accordingly, we detected MEF2-binding consensus sequences (C/T)TA(A/T)_4_TA(G/A) within the *SOD3* promoter region, suggesting that MEF2 plays a critical role in the regulation of SOD3.

In the present study, we investigated whether a treatment with CAPE significantly induces the expression of SOD3 in HRECs. The results obtained showed for the first time that CAPE-elicited SOD3 expression was mediated by histone H3 acetylation within the *SOD3* promoter region, and this was attributed to a weaker MEF2A/D-HDAC1 interaction.

## Materials and Methods

### Reagents

HRECs and CSC complete recombinant medium were purchased from DS Pharma Biomedical Co. (Osaka, Japan). CAPE was purchased from Wako Pure Chemicals (Osaka, Japan). Anti­acetyl­histone H3 (#06-599), anti-acetyl-histone H4 (#06-598) rabbit polyclonal, and anti­actin mouse monoclonal (MAB1501) antibodies were purchased from Millipore Co. (Billerica, MA). Anti-HDAC1 (sc-7872), -HDAC3 (sc-11417) rabbit polyclonal antibodies and anti-MEF2 (sc-313), anti-MEF2X (sc-313X) rabbit polyclonal antibodies were purchased from Santa Cruz Biotechnology, Inc. (Dallas, TX). An anti-HDAC1 (#5356) mouse monoclonal antibody and normal rabbit IgG (#2729) were purchased from Cell Signaling Technology (Danvers, MA). An anti-MEF2D (610774) mouse monoclonal antibody was purchased from BD Transduction Laboratories (Lexington, KY). Horseradish peroxidase (HRP)-conjugated anti­rabbit (A6154) or ­mouse (A4416) IgG (whole molecule)-peroxidase antibodies were purchased from Sigma­Aldrich, Inc. (Saint Louis, MO). An anti-MEF2A phospho T312 (ab30644) rabbit polyclonal antibody was purchased from abcam (Cambridge, UK).

### Cell culture

HRECs were cultured with CSC complete recombinant medium containing 100 units/ml penicillin and 100 µg/ml streptomycin in coating dishes by 10% Cell matrix Type I-C (Nitta Gelatin, Osaka, Japan) at 37°C in a humidified 5% CO_2_ incubator. HRECs were split at 90% confluence, and culture media were changed every 2 days.

### Cytotoxicity assay

The lactate dehydrogenase (LDH) assay was used to estimate cytotoxicity. HRECs were treated with CAPE, H_2_O_2_ or 6-hydorxydopamine (6-OHDA) in a 96-well micro plate. After treatment, LDH released into conditioned medium was analyzed using a LDH cytotoxic test (Wako Pure Chemicals) according to manufacture’s protocol.

### Reverse transcription­polymerase chain reaction (RT­PCR) analysis

HRECs were treated with CAPE in a 60-mm culture dish. After the treatment, cells were washed with cold phosphate-buffered saline (PBS) and total RNA was extracted from cells with 1 ml of TRIzol reagent (Invitrogen, Carlsbad, CA). The synthesis of cDNA was performed using the ReverTra Ace qPCR RT Kit (Toyobo, Osaka, Japan) according to the manufacturer’s protocol. RT-PCR for the expression of SODs was performed using our previously described method.^(28)^ RT-PCR was performed in TaKaRa PCR Thermal Cycler Dice Gradient using the following parameters: 94°C denaturation for 2 min followed by performing PCR response on a condition to show in Table 1. SOD3 were amplified with 0.4 mM dNTP, 12 pmol sense primer, 12 pmol antisense primer and 0.4 unit KOD Fx. Other genes were amplified with 0.2 mM dNTP, 10 pmol sense primer, 10 pmol antisense primer and 0.4 unit KOD Fx. The primer sequences used in RT-PCR are shown in Table 1. After amplification, aliquots of the PCR mixtures were separated on a 2% (w/v) agarose gel and stained with ethidium bromide. A densitometric analysis of the PCR products was performed with Multi Gauge V 3.0 (Fuji Film, Tokyo, Japan). mRNA levels were normalized to those of 18S rRNA in each sample.

Real-time RT-PCR for the expression of HDACs was performed using the Thunderbird SYBR qPCR Mix (Toyobo) and in Thermal Cycler Dice Real Time System II using the following parameters: 95°C denaturation for 1 min followed by 50 cycles of 95°C for 15 s, 60°C for 1 min and dissociation at 95°C for 15 s, 60°C for 30 s and 95°C for 15 s. mRNA levels were normalized to those of 18S rRNA in each sample. The primer sequences used in RT-PCR are shown in Table 2.

### Western Blotting

Nuclear fractions and core histones were prepared from cells as described in our previous study.^(29,30)^ Isolated histones or nuclear extracts containing 20 µg protein were boiled with sodium dodecyl sulfate (SDS) buffer (3% SDS, 10% glycerol, 62.5 mM Tris, pH 6.8) and 5% 2-mercaptoethanol for 5 min, and were then separated by SDS-PAGE on 15 or 10% (w/v) polyacrylamide gels, followed by their transfer electrophoretically onto PVDF membranes. Non-specific binding sites were blocked with PBS containing 1% bovine serum albumin (BSA). The membranes were then incubated with the respective specific primary antibodies (1:1,000) followed by incubation with the biotin-conjugated goat anti-rabbit or -mouse IgG antibody (1:1,000). Bands were detected using SuperSignal West Pico (Thermo Scientific, Rockford, IL) and imaged using LAS-3000 UV mini (Fuji Film).

### Chromatin immunoprecipitation (ChIP) assay

ChIP assays were performed as described in our previous study with minor modifications.^(29)^ Sheared genomic DNA was immunoprecipitated with primary antibodies overnight, and this was followed by an incubation with Dynabeads Protein G (Invitrogen) for 2 h. The abundance of *SOD3* promoter regions in ChIP precipitates was quantified using a PCR analysis. The primer sequences used in the ChIP assay were as follows: sense 5'-GTG GAG GCG AAG CAA TTC TA-3', antisense 5'-CTG TTA GCG CGA GTG CAG GA-3' (126 bp). After amplification, these PCR products were loaded onto a 2% (w/v) agarose gel for electrophoresis and visualized using FLA5100. A densitometric analysis of the PCR products was performed with Multi Gauge V3.0.

### Immunoprecipitation (IP)

A whole cell extract was prepared from HRECs as described below. After cells had been treated with CAPE, they were collected and lysed in 1 ml of lysis buffer [20 mM Tris-HCl, pH 7.5, 150 mM NaCl, 1 mM EDTA, 1 mM EGTA, 1% Triton X-100, 10 mM NaF, 1 mM Na_3_VO_4_, 20 mM β­glycerophosphate, 5 µg/ml leupeptin, 1 mM dithiothreitol (DTT), and 1 mM phenylmethylsulfonyl fluoride (PMSF)]. After homogenization by an ultrasonic homogenizer (Mitsui Electric Co., Chiba, Japan), they were stirred at 4°C for 30 min. Cells were then centrifuged at 18,000 × *g* for 10 min and the protein concentration of the resulting supernatant was measured with the Bio-Rad Protein Assay (Bradford, Hercules). Extracts containing 500 µg protein were incubated with the respective primary antibodies (1 µg) at 4°C overnight. The solution was then incubated with 20 µl of Dynabeads Protein G for 2 h. After the incubation, beads were sequentially washed with lysis buffer and PBS twice for each solution, and then incubated in SDS buffer at 70°C for 10 min and at 95°C for 5 min after the addition of 5% 2-mercaptoethanol. Samples were separated by SDS-PAGE on 10% (w/v) polyacrylamide gels. After being transferred electrophoretically onto PVDF membranes, non-specific binding sites were blocked with 5% skim milk in PBS containing 0.1% Tween 20 (PBST). The membranes were then incubated with the respective primary antibodies (1:2,000), and this was followed by an incubation with the anti-mouse IgG (whole molecule)-peroxidase antibody (1:5,000). After the membranes had been washed three times with PBST, the bands were detected using SuperSignal West Pico, and imaged using LAS-3000 UV mini.

### Statistical analysis

Data are expressed as the means ± SE of three independent experiments. Statistical evaluations of the data obtained were performed using ANOVA followed by post-hoc Bonferroni tests. A *p* value less than 0.05 was considered significant.

## Results

### Effect of CAPE on oxidative stress-triggered HRECs injury

Treatment with 100 µM H_2_O_2_ or 50 µM 6-OHDA induced HRECs injury, and pretreatment with 10 µM CAPE some, but significantly, suppressed these injuries (Fig. 1B).

### Effect of CAPE on the expression of SODs in HRECs

**Fig. 2:**
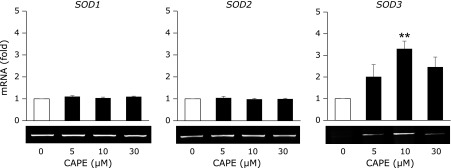
Effects of CAPE on the expression of SODs. HRECs were treated with the indicated concentrations of CAPE for 24 h. After cells had been treated, the level of each mRNA was measured by RT-PCR. RT-PCR data were normalized using 18S rRNA levels. Data were shown as the mean ± SE (*n* = 3). **p<0.01 vs untreated cells.

The treatment of HRECs with CAPE for 24 h significantly increased mRNA level of SOD3 at the concentration of 10 µM, but not those of SOD1 and SOD2 (Fig. 2). Moreover, we evaluated the effect of CAPE on the expressions of antioxidant enzymes, such as catalase and glutathione peroxidase, but these expressions were also not induced (data not shown).

### Effect of CAPE on the levels of acetylated histone within* SOD3* promoter region

**Fig. 3:**
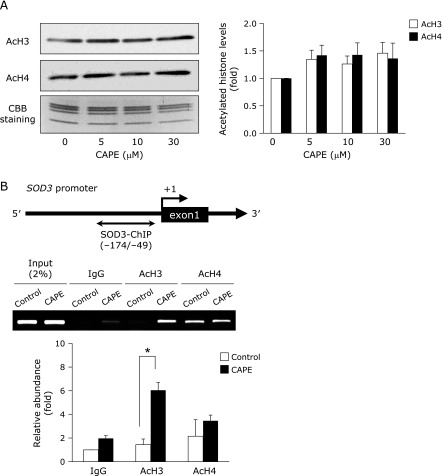
Effects of CAPE on acetylated histone levels within the *SOD3* promoter region. (A) HRECs were treated with the indicated concentrations of CAPE for 24 h. After cells had been treated, AcH3 and AcH4 were measured by Western blotting. The loading amount of histone was monitored by Coomassie Brilliant Blue staining (CBB). Values are the means of fold changes from vehicle-treated cells (*n* = 3). (B) Schematic representation of the locations of PCR primers used in the ChIP assay to amplify the *SOD3* promoter. Nucleotide positions were numbered relative to the major transcriptional start site (+1). HRECs were treated with or without 10 µM CAPE for 24 h. After cells had been treated, the ChIP assay was performed. Relative binding to the promoter region was expressed as a fold amount over input (2%). Data were shown as the mean ± SE (*n* = 4). *p<0.05.

HRECs were treated with CAPE for 24 h and acetylated histone H3 or H4 were determined by Western blotting, but CAPE did not induce the acetylated levels of histone H3 and H4 (Fig. 3A). Therefore, we next performed ChIP assays to investigate whether CAPE induces the histone acetylation within the proximal promoter region of *SOD3*. As expected, the treatment with 10 µM CAPE enhanced the levels of acetylated histone H3 but not H4 within the *SOD3* promoter region (Fig. 3B).

### Effect of CAPE on the expression of HDACs

**Fig. 4:**
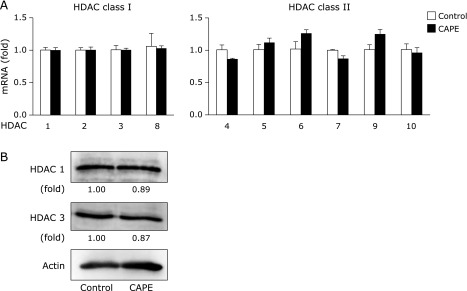
Effects of CAPE on the expression of HDACs. (A) HRECs were treated with 10 µM CAPE for 24 h. After cells had been treated, mRNA levels were measured by real-time RT-PCR. Real-time RT-PCR data were normalized using 18S rRNA levels. Data were shown as the mean ± SE (*n* = 3). (B) HRECs were treated with 10 µM CAPE for 24 h. After cells had been treated, the protein expression of HDAC1 and HDAC3 was detected by Western blotting. Values are expressed as fold change relative to the level of HDAC1 or HDAC3 in control cells.

We next investigated the possibility that CAPE functions as a HDACs inhibitor in HRECs, because CAPE is structurally similar to suberoylanilide hydroxamic acid and trichostatin A (TSA), class I and II of HDACs inhibitors. As shown in Fig. 4A and B, the expressions of HDAC classes I and II mRNA and HDAC 1 and 3 proteins in nuclear were not changed by the treatment with CAPE. Moreover, we examined the effect of CAPE on the expression of HDAC 9, a class II HDACs; however, treatment with CAPE did not affect the expression (data not shown).

### Effect of CAPE on the interaction of MEF2A and HDAC1

**Fig. 5:**
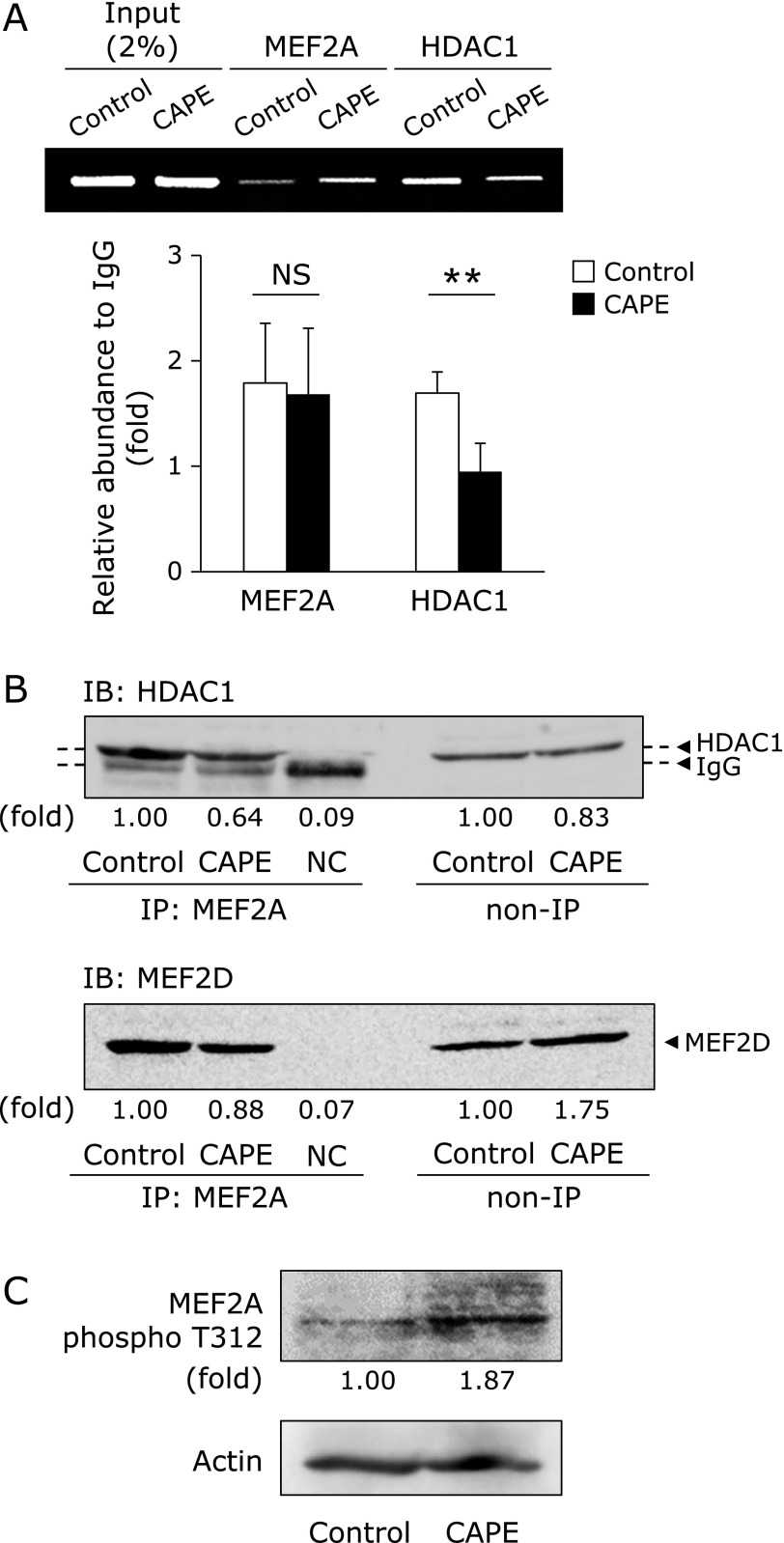
Effects of CAPE on the interaction between MEF2A and HDAC1. (A) HRECs were treated with 10 µM CAPE for 24 h. After cells had been treated, the ChIP assay was performed. Relative binding to the promoter region was expressed as a fold amount over input (2%). Data were shown as the mean ± SE (*n* = 4). **p<0.01, NS, not significant. (B) HRECs were treated with 10 µM CAPE for 24 h. After cells had been treated, the interaction between MEF2A and HDAC1 or MEF2D was assessed by IP. NC, negative control. (C) The phosphorylation of MEF2A threonine 312 (MEF2A phospho T312) was determined by Western blotting. Values (B and C) are expressed as fold change relative to the level of HDAC1 or MEF2D (B), or MEF2A phospho T312 (C) in control cells.

We tried to search for the DNA binding protein that is possible to bind to *SOD3* promoter region. *In silico* analysis of putative DNA binding site in the *SOD3* promoter revealed the possibility that MEF2 binds to the *SOD3* promoter and functions as a key DNA binding protein. Therefore, we speculated that CAPE-elicited SOD3 expression is due to the dissociation of HDAC1 from MEF2A/D complex within the *SOD3* promoter region. As expected, our ChIP results clearly demonstrated that treatment with CAPE decreased the enrichment of HDAC1 within its region (Fig. 5A). Furthermore, treatment with CAPE dissociated MEF2A and 2D interaction, indicating that the complex of MEF2A/D-HDAC1 plays a significant role in SOD3 silencing in HRECs (Fig. 5B). Moreover, treatment with CAPE increased the phosphorylation of the 312 threonine residue in MEF2A (Fig. 5C).

## Discussion

Impaired redox homeostasis results in the induction of oxidative stress;^(31)^ however, a certain level of oxidative stress is necessary for normal metabolic processes because reactive oxygen species (ROS) have a number of regulatory roles in cells.^(32)^ Oxidative stress-related cell injury has been reported under hyperglycemic conditions and has been implicated in the pathology of DR. Accordingly, the maintenance of anti-oxidative activities suppresses the pathogenesis of DR. We previously proposed that SOD3 plays a critical role in suppressing the pathogenesis of DR.^(5,7)^ Moreover, the expression of SOD3 is known to be epigenetically silenced in HRECs, and a treatment with exendin-4 induced its expression through histone acetylation,^(17)^ suggesting that reagents exerting epigenetic effects maintain redox homeostasis by regulating the expression of SOD3.

Previous studies revealed that natural products with catechol rings possess anti-oxidative properties,^(33)^ and we also demonstrated that luteolin, a flavone, significantly suppressed oxidative stress-related events.^(34,35)^ It has been reported that CAPE is decomposed into caffeic acid and phenethyl alcohol by intracellular esterase, and caffeic acid with catechol skeleton possess anti-oxidative properties.^(8,36)^ We recently reported that a treatment with CAPE suppressed monocyte adhesion to endothelial cells by inhibiting the accumulation of intracellular ROS.^(37)^ In this study, we determined that pretreatment with CAPE significantly suppressed H_2_O_2_ or 6-OHDA-triggered HREC cell injury (Fig. 1B). Our RT-PCR results showed that the treatment with CAPE significantly increased SOD3 mRNA levels (Fig. 2), and these results are consistent with previous findings showing that exendin-4 selectively induced the expression of SOD3.^(17)^ SOD3 is the only SOD isozyme present in extracellular spaces, attaches to the cell surface via its heparin-binding domain, and protects endothelial cells from extracellularly generated superoxide.^(38–40)^ However, SOD3 expression levels are very low in HRECs.^(17)^ Furthermore, previous studies reported that SOD3 activity was decreased with type 2 diabetes and its associated arteriosclerosis.^(6,41,42)^ Accordingly, the up-regulation of SOD3 expression in endothelial cells is considered to be of significant importance for suppressing the development of DR.

Accumulated evidence has revealed that the expression of SOD3 is regulated by epigenetic mechanisms,^(31,39–41)^ including DNA methylation and histone modifications. We recently revealed that the up-regulation of SOD3 expression in HRECs depended on histone acetylation through the inhibition of HDACs, and not on DNA methylation.^(19)^ The proximal promoter regions of *SOD3*, at least those from –173 to –35, in HRECs are not methylated, and its expression was unchanged by a treatment with 5-azacytidine, a DNA methyltransferase inhibitor.^(17)^ On the other hand, this study demonstrated that the expression of SOD3 in HRECs was induced by histone acetylation based on the findings of a treatment with TSA and also valproic acid up-regulating its expression in a concentration-dependent manner.^(17)^ In this study, treatment with CAPE did not affect the acetylated levels of histone H3 or H4, but significant enrichment of acetylated histone H3 within the proximal promoter region of *SOD3* (Fig. 3B). Recently, it was reported that CAPE functions as directly or indirectly inhibitor of HDAC and is a naturally occurring epigenetic therapeutic agent.^(10)^ However, as shown in Fig. 4A, the expressions of HDAC classes I and II mRNA and HDAC 1 and 3 proteins in nuclear were not changed by the treatment with CAPE. Moreover, we examined the effect of CAPE on the expression of HDAC 9, a class II HDACs; however, treatment with CAPE did not affect the expression (data not shown). These observations suggested that the changes of interaction with HDAC and scaffold protein, which directly binds to the *SOD3* promoter, may play an important role in CAPE-elicited SOD3 expression.

It is well known that MEF2 proteins are expressed in distinct, but overlapping patterns in embryonic and adult tissues.^(43)^ Furthermore, MEF2 functions as a homo- or heterodimer and binds to the consensus DNA sequence (C/T)TA(A/T)_4_TA(G/A).^(20,44–49)^ MEF2 has also been reported to interact with chromatin modifiers, including HDAC4, HDAC5, HDAC7, and HDAC9 or p300 in muscle cells or lymphocytes.^(20,22–26,44–52,54)^ Among the MEF2 proteins tested, MEF2A was enriched the most within the proximal promoter region of *SOD3* in HRECs, suggesting that it is a key scaffold protein in CAPE-elicited SOD3 expression. A recent study reported that the MEF2A/D heterodimer strongly interacted with HDAC1, and this interaction was dissociated during macrophage differentiation, leading to the induction of c-jun.^(43)^ Our ChIP results clearly demonstrated that the treatment with CAPE decreased the enrichment of HDAC1 within its region (Fig. 5A). Furthermore, the treatment with CAPE dissociated the MEF2A and 2D interaction, indicating that the MEF2A/D-HDAC1 complex plays a significant role in SOD3 silencing in HRECs (Fig. 5B). It has been reported that the phosphorylation of the 312 threonine residue in the C-terminal region of MEF2A is involved in the transcriptional activation of MEF2A.^(55,56)^ In this study, the treatment with CAPE increased the phosphorylated MEF2A T312 level (Fig. 5C). Based on these results, the phosphorylation of MEF2A by the CAPE treatment promoted the dissociation of MEF2A/2D-HDAC1 complex, and which leads to SOD3 induction by acetylating of histone H3 within the proximal *SOD3* promoter.

**Fig. 6:**
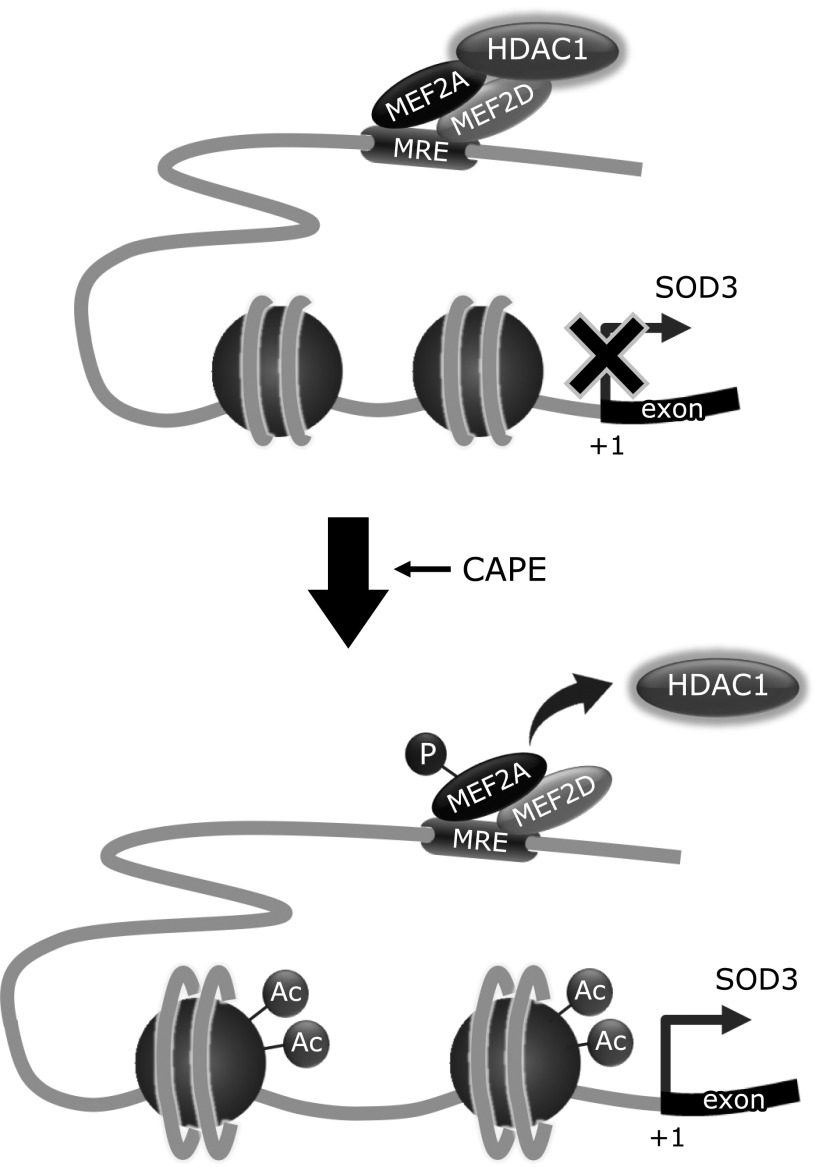
Proposed model for the involvement of MEF2A/D and HDAC1 in the CAPE-induced up-regulation of SOD3 expression. Under basal conditions, MEF2A/D-HDAC1 complexes bind to the MEF2 regulatory element (MRE) within the *SOD3* promoter region, and inhibit the transcription of SOD3 through histone deacetylation. After HRECs have been treated with CAPE, HDAC1 dissociates from the MEF2A/D heterodimer, which increases the enrichment of acetylated histone H3 within the *SOD3* promoter region and induces its expression.

In conclusion, the present study demonstrated that the CAPE-induced up-regulation of SOD3 expression through acetylated histone H3 was a result of the dissociation of HDAC1 within the *SOD3* promoter region. We are the first to show that the MEF2A/D heterodimer functions as a key scaffold protein in the regulation of SOD3 expression (Fig. 6). The up-regulated expression of SOD3 on the endothelial cell surface induces a delay in the development of microvascular impairments. Although our present data might suggest that CAPE could be one of seed compounds to maintain the redox homeostasis, it is necessary to consider how CAPE applies to clinical usage. Recently, it was reported that the solubility of CAPE was improved by composing with γ-cyclodextrin and this enhanced its bioavailability.^(57)^ Moreover, it should be discussed about drug delivery system for effectively delivering CAPE to retinal endothelial cells. It currently remains insufficient about above problems; however, a better understanding of the role of epigenetics in redox homeostasis, and a developing the clinical application of CAPE may delay the progression of DR.

## Figures and Tables

**Table 1:** Primer sequences used in RT-PCR and PCR conditions

Primer		Sequence (5' to 3')	Cycle	Annealing (s)	Elongation (s)
SOD 1	forward	GCGACGAAGGCCGTGTGCGTG	30	60°C (40)	72°C (60)
	reverse	TGTGCGGCCAATGATGCAATG			

SOD 2	forward	CGACCTGCCCTACGACTACGG	30	60°C (40)	72°C (60)
	reverse	CAAGCCAACCCCAACCTGAGC			

SOD 3	forward	AGAAAGCTCTCTTGGAGGAG	33	60°C (30)	68°C (60)
	reverse	ACCGCGAAGTTGCCGAAGTC			

18S rRNA	forward	CGGCTACCACATCCAAGGAA	15	60°C (45)	72°C (45)
	reverse	GCTGGAATTACCGCGGCT			

**Table 2:** Primer sequences used in real-time RT-PCR

Primer		Sequence (5' to 3')
HDAC 1	forward	CCTGAGGAGAGTGGCGATGA
	reverse	GTTTGTCAGAGGAGCAGATCGA

HDAC 2	forward	GCTCTCAATGGCGGTTCAG
	reverse	AGCCCAATTAACAGCCATATCAG

HDAC 3	forward	CCCAGACTTCACACTTCATCCA
	reverse	GGTCCAGATACTGGCGTGAGTT

HDAC 4	forward	GACCTGACCGCCATTTGC
	reverse	GGGAGAGGATCAAGCTCGTTT

HDAC 5	forward	CAACGAGTCGGATGGGATGT
	reverse	GGGATGCTGTGCAGAGAAGTC

HDAC 6	forward	TGCCTCTGGGATGACAGCTT
	reverse	CCTGGATCAGTTGCTCCTTGA

HDAC 7	forward	AGCAGCTTTTTGCCTCCTGTT
	reverse	TCTTGCGCAGAGGGAAGTG

HDAC 8	forward	CGGCCAGACCGCAATG
	reverse	CACATGCTTCAGATTCCCTTT

HDAC 9	forward	AGGCTCTCCTGCAGCATTTATT
	reverse	AAGGGAACTCCACCAGCTACAA

HDAC 10	forward	ATGACCCCAGCGTCCTTTACT
	reverse	CGCAGGAAAGGCCAGAAG

18S rRNA	forward	CGGCTACCACATCCAAGGAA
	reverse	GCTGGAATTACCGCGGCT

## Abbreviations

CAPE: caffeic acid phenethyl ester
ChIP: chromatin immunoprecipitation
DR: diabetic retinopathy
EC-SOD: extracellular superoxide dismutase
HAT: histone acetyltransferase
HDAC: histone deacetylase
HRECs: human retinal endothelial cells
LDH: lactate dehydrogenase
MEF2: myocyte enhancer factor 2
MRE: MEF2 regulatory element
6-OHDA: 6-hydroxydopamine
PBS: phosphate-buffered saline
ROS: reactive oxygen species
RT-PCR: reverse transcription-polymerase chain reaction
TSA: trichostatin A
